# Reactivation of tuberculosis in cancer patients following administration of immune checkpoint inhibitors: current evidence and clinical practice recommendations

**DOI:** 10.1186/s40425-019-0717-7

**Published:** 2019-09-04

**Authors:** Amalia Anastasopoulou, Dimitrios C. Ziogas, Michael Samarkos, John M. Kirkwood, Helen Gogas

**Affiliations:** 1 First Department of Medicine, Laiko General Hospital, National and Kapodistrian University of Athens School of Medicine, 11527 Athens, Greece; 2 Division of Hematology/Oncology, University of Pittsburgh School of Medicine, Pittsburgh, PA 15213 USA

**Keywords:** Immunotherapy, Cancer, Immune checkpoint inhibitors, Tuberculosis

## Abstract

Immune checkpoint inhibitors (ICBs) have revolutionized cancer treatment producing remarkable and durable responses for a range of malignancies. However, the additional modulation of immune response by ICBs may rarely cause immune-related infectious complications, including re-activation of latent tuberculosis infection (LTBC) with detrimental effects on those patients’ outcome. Here, we present two “real-world” melanoma cases that were treated in our department with blockade of PD-1/PD-L1 and developed active *Mycobacterium tuberculosis* (MTB) during immunotherapy. In view of these cases, we review the literature for ICB-associated MTB reactivation and discuss our considerations about the possible interactions of immunotherapy and the underlying co-existent mycobacterial infection. Based on the current evidence from preclinical findings prior to this experience, we raise questions regarding cancer patients who are at higher risk for developing MTB infection, whether ICB-treated patients should be considered immunocompromised, and how they should be managed for latent and/or active tuberculosis. Aside from the well-established clinical benefit of immunotherapy, the blockade of PD-1/PD-L1 axis may concurrently disrupt the immune control of specific opportunistic infections such as tuberculosis that should be carefully and expectantly managed in order to avoid compromising the outcome of cancer treatment and the affected patient’s survival.

## Introduction

The development of immune checkpoint inhibitors (ICBs) has radically changed the way that numerous cancers are treated. After the initial approval of ipilimumab (a monoclonal antibody blocking CTLA-4) for the treatment of metastatic melanoma, five more antibodies that target PD-1/PD-L1 pathways, including nivolumab and pembrolizumab (against PD-1) and atezolizumab, avelumab, and durvalumab (against PD-L1), have been licensed and introduced into the therapeutic algorithms for various malignancies in first and later lines of treatment, as well as in the neoadjuvant and adjuvant settings [1]. Cancer cells are able to evade host immune surveillance and escape tumor neutralization by inhibiting PD-1 targeted cancer-specific T cells via overexpression of PD-L1 [2]. The monoclonal antibodies prevents the binding of PD-1 to its ligand PD-L1, restores the T cell-mediated cytotoxicity and allows the immune natural defense to fight against cancer with significant clinical benefits [3]. However, the immune stimulation triggered by these drugs may lead to severe and even life-threatening, albeit infrequent, immune-related adverse events (irAEs) involving almost every organ [4, 5]. Current guidelines on the management of irAEs recommend the prompt administration of high-dose corticosteroids and if toxicity persists, further immunosuppression with steroid-sparing regimens (e.g. anti-tumor necrosis factor-alpha (TNF-a) agents or mycophenolate mofetil) [6].

In this context, the additional modulation of immune response due to the cancer itself, due to the administration of ICPIs or the supplementary medications (e.g. steroids or anti-TNF agents) for overcoming irAEs may unmask chronic underlying or opportunistic infections and rarely cause some serious infectious complications such as varicella-zoster virus infection, cytomegalovirus-associated enterocolitis, pulmonary aspergillosis, pneumocystis pneumonia and reactivation of latent tuberculosis with detrimental, in some cases, effects on cancer treatment outcome and patient’s survival [7, 8]. The overall incidence of these serious immune-related infections in 740 patients with metastatic melanoma receiving ICBs was estimated to be 7.3% in a recent review, where infectious complications were detected mainly in patients who required corticosteroids and/or TNF-a inhibitors [9].

Given the high incidence of *Mycobacterium tuberculosis* (MTB) infection worldwide and the poor prognosis of MTB reactivation, a renewed interest was developed to recognize individuals at high risk that should be screened for early detection of latent tuberculosis and treated to prevent active disease [10, 11]. The Center for Disease Control and Prevention (CDC), the World Health Organization (WHO), and the US Preventive Services Task Force (USPSTF) agree that the risk of exposure to MTB is higher: a) in patients living or working in endemic countries (e.g. East Asia and Central America) and b) in patients living in large group settings (e.g. homeless or military shelters and prisons). In most patients infected with MTB, the disease remains clinically asymptomatic and inactive, however, in 5–10% of them, the infection will reactivate at some point during their lifetime with a baseline risk between 6 and 20 per 100,000 person-years [12]. After that, the risk of reactivation depends on the specific type of immunosuppression [11, 13]. Compared to the general population, this risk is greater among solid organ transplant recipients (15xfold) [14] and stem cell transplant recipients (8-12xfold) [15], followed by patients treated with anti-TNF medications (5-7xfold) [16–19], while in patients with HIV infection, it reaches 50 times higher and causes up to 25% of deaths among patients [20]. Other host factors that may increase the susceptibility to develop active tuberculosis include older age (> 60 years), prior tuberculosis history, chronic obstructive pulmonary disease, heavy smoking or increased alcohol consumption, diabetes mellitus or end-stage renal disease and for these patients screening is also recommended [13, 21–23]. Cancer has been recognized as an independent risk factor for developing active MTB infection since the 1970s, however this risk widely varies among cancer types, is differentially affected by modern therapies (targeted agents and monoclonal antibodies) and remains to be precisely quantified.

In this study, we present two melanoma patients who developed active tuberculosis during their treatment with PD-1/PD-L1 blockade in our department. In view of these two cases, we review the literature from the preclinical data on the immune-mediated interactions of PD-1/PD-L1 inhibition and co-existent tuberculosis, and published clinical reports with ICB-associated tuberculosis. Integrating the current evidence with our institutional experience, we address questions about which cancer patients are at higher risk for MTB infection, whether ICB-treated cases should be still considered immunocompromised, and how they should be managed for latent or active tuberculosis.

## Case 1

**Fig. 1:**
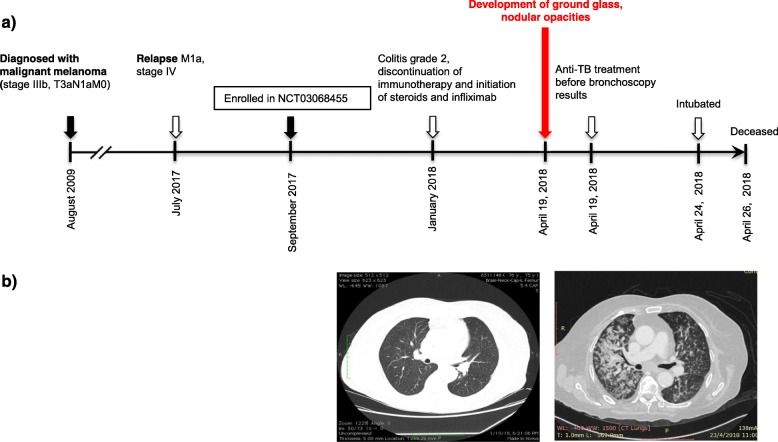
Development of active MTB in a patient treated with nivolumab+/-ipilimumab for metastatic melanoma in the setting of a clinical trial. **a** Timeline of therapy and disease status for both melanoma and TB. **b** Chest CT images of patient at the enrollment in the clinical trial before the initiation of PD-1 inhibition (15 October 2016, left) and ~20 weeks later (19 April 2018, right)

A 76-year-old Greek woman was diagnosed with a cutaneous melanoma lesion of her left lower leg in August 2009 (Fig. 1). Her comorbidities included smoking of 45 pack*years, hypertension, dyslipidemia, coronary artery disease and osteopenia. She underwent a radical resection of the tumour, but the sentinel lymph node was grossly infiltrated (stage IIIb, T3aN1aM0), and she received interferon (IFN) 20,000 iu/m^2^ every day during December 2009, according to contemporary recommendations. She remained disease free until July 2017 when she developed a new cutaneous lesion of her left calf (M1a, stage IV). PET/CT scanning did not show other distant metastasis. For her metastatic recurrent melanoma, the patient enrolled in a clinical trial (ClinicalTrials.gov ID: NCT03068455) and was randomized to receive monotherapy with nivolumab 240 mg every 2 weeks versus the combination of nivolumab with ipilimumab 1 mg/kg every 3 weeks. Due to her smoking history, she was under regular follow-up by pulmonologist and had a negative tuberculin skin test (TST) on March 2017 but the trial protocol did not require LTBC screening before the initiation of immunotherapy. In January 2018, after 8 doses of immunotherapy, she presented diarrhea grade 2 and started methylprednisolone 16 mg po twice daily with a slow taper (over 4–6 weeks). After a short-term improvement of her diarrhea to grade 1, her symptoms worsened again and a colonoscopy was performed. The endoscopic examination revealed grade 3 colitis with multiple ulcerative mucosal lesions. Therefore, immunotherapy was permanently discontinued and the dose of methylprednisolone was increased to 32 mg daily and intravenous (iv) infliximab administered at a dose of 5 mg/kg. After three doses of infliximab her colitis improved (to grade 1) and steroid taper was resumed. Two weeks later, the patient was admitted to our hospital with fever up to 38 °C, fatigue and weight loss. Physical examination suggested a lower respiratory tract infection. Laboratory tests revealed neutrophils: 6700/μL, hemoglobin: 10.5 g/dL, platelet count: 129,000/μL and elevated C reactive protein (CRP = 147.6 mg/L). The patient was empirically treated with iv tazobactam/piperacillin 4.5 mg 4 times daily. Computed tomography (CT) scanning of the chest showed interval development of ground glass and centrilobular nodular opacities predominantly in the right lung (Fig. 1). These imaging findings were not present in a prior CT scan 2 months before. On April 19, 2018, the patient underwent bronchoscopy and was started on anti-tuberculosis medication with rifampin 600 mg/day, isoniazid 300 mg/day and ethambutol 1200 mg/day and pyrazinamide 2000 mg/day as well as anti-pneumocystis treatment with trimethoprim-sulfamethoxazole 20 mg/kg/day. The polymerase chain reaction (PCR) of bronchoalveolar lavage (BAL) was positive for MTB complex. Despite immediate treatment, her respiratory function was progressively worsened and the patient was moved to intensive care unit. She was intubated but remained persistently febrile and hypotensive requiring vasopressor medications. The patient passed away 2 days later and subsequently the BAL culture grew MTB with no resistance to anti-tuberculosis treatment, according to susceptibility testing.

## Case 2

**Fig. 2:**
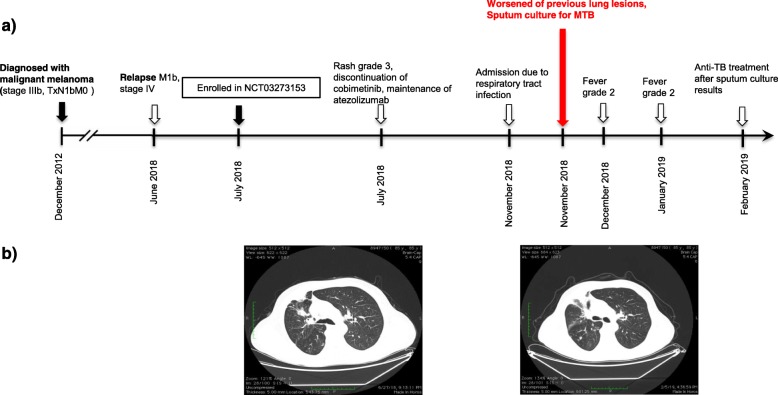
Development of active MTB in a patient treated with atezolizumab and cobimetinib for metastatic melanoma in the setting of a clinical trial. **a** Timeline of therapy and disease status for both melanoma and MTB. **b** Chest CT images of patient at the enrollment in the clinical trial (July 2018, left) and 4 months later (November 2018, right)

An 85-year-old Greek man was diagnosed with melanoma of the right parotid nodes confirmed by positive fine needle aspiration on December 2012 (Fig. 2). His medical history included hypertension, dyslipidemia, glaucoma and benign prostate hyperplasia. The patient underwent a total parotidectomy and regional lymph node dissection as well as a tonsil biopsy. Both right parotid gland and dissected lymph nodes were infiltrated by melanoma, while biopsy of tonsil was negative. No primary skin lesion was recognized and subsequent staging scans were also negative for residual disease (stage IIIb, TxN1bM0). Therefore, he received prophylaxis with high dose IFN 20000 iu/m^2^ every day and he was followed-up until June 2018. At this time, he underwent a chest CT because of persistent cough, which revealed multiple lymph nodes and a mediastinal soft tissue mass (M1b, stage IV) (Fig. 2). In the context of a clinical trial (ClinicalTrials.gov ID: NCT03273153), he started a combination with atezolizumab 840 mg every 3 weeks and MEK inhibitor cobimetinib 60 mg every day for his metastatic BRAFV600 wild-type melanoma. The patient was not tested for LTBC before starting anti-melanoma treatment, because he had no known risk factors for MTB reactivation and the clinical trial protocol did not require it. After receiving the therapeutic combination for approximately 5 months, including a temporary interruption of cobimetinib because of grade 3 rash, the patient presented with symptoms of lower respiratory tract infection grade 3 on November 2018. No new imaging findings by X-ray were recognized at that time. Cobimetinib was interrupted again and the patient received a course of iv tazobactam/piperacillin 4.5 mg QID and levofloxacin 500 mg every day. During the next 3 months, the patient had two more episodes of fever grade 2, during which he was hospitalized and received broad-spectrum antibiotics for 1 week in total each time. Throughout this period, cobimetinib was temporarily interrupted, but immunotherapy with atezolizumab was continued without complications. Although acid-fast bacilli stain of sputum was negative, a sputum culture taken in his last hospitalization grew M. tuberculosis. Susceptibility testing showed susceptibility to all antimycobacterial agents. On February 2019, he initiated a 3-drug regimen, including isoniazid 300 mg/day, rifampin 600 mg/day and pyrazinamide 1500 mg/day. Currently, he continues anti-tuberculosis medication with good tolerability, whereas anti-melanoma combination is still withheld up to resolution of MTB imaging lesions.

## Preclinical data in mice and human cells

Although T-helper type 1 CD4+ cells (Th1) are required for control of mycobacterial infections, the enhanced CD4 activity in the absence of PD-1 surveillance exacerbates tuberculosis in mouse models. This status of T cell exhaustion arises from the sustained activation with absence of inhibitory receptors and prevents optimal control of infection and tumors [24]. In fact, PD-1 knockout (PD-1−/−) mice are more susceptible to MTB mortality, developing large necrotic lesions with high bacterial loads and succumbing faster than even T cell-deficient mice [25–27]. The inability of PD-1−/− mice to control mycobacterial infection is attributed to increased Th1-mediated responses and overproduction of interferon-gamma (IFN-γ) [26]. Sakai et al. pointed out that activated PD-1/PD-L1 signaling suppresses the accumulation of parenchymal CD4+ T cells and limits IFN-γ production, protecting mice from fatal exacerbated pulmonary mycobacterial infection [28]. Cancer cells and infectious agents may evade early immune responses with other PD-1/PD-L1 mediated mechanisms: i) promotion of PD-L1 expression on dendritic cells and enhanced induction of Treg cells [29, 30], ii) overexpression of PD-1 on NK cells, as detected in patients with multiple myeloma [31] or infected with MTB [32] or HIV [33]. A recent study by Cao et al. [34] found that co-stimulation by MTB and lung cancer antigen in mice may partially reverse the loss of T-cell function via PD-1/PD-L1 pathway and prevent the rapid evolution of advanced lung cancer [34].

In human patients with active tuberculosis, PD-1 was increased on CD4+ T cells but not on CD8+ T cells compared to healthy controls [35, 36] while effective anti-tuberculosis treatment was associated with a down-regulation of PD-1 on CD4+ T cells [36]. Similarly, the expressions of PD-1 and PD-L1 on monocytes from patients with active MTB were much higher compared to healthy controls, while phagocytosis and intracellular killing activity of macrophages increased significantly with PD-1/PD-L1 blockade in vitro [35]. In a patient with Merkel cell carcinoma treated with nivolumab, IFN-γ-producing MTB-specific CD4+ T cells were detected in the blood months before the development of a tuberculoma [37]. Producing a scenario similar to immune reconstitution inflammatory syndrome (IRIS), the blockade of PD-1 axis boosts Th1-mediated inflammatory responses and causes a worsening of damage to the MTB-infected tissue [38]. Currently, the PD-1/PD-L1 pathway is also being studied as a novel host-directed target in multidrug-resistant tuberculosis [39, 40].

## Literature search

**Fig. 3:**
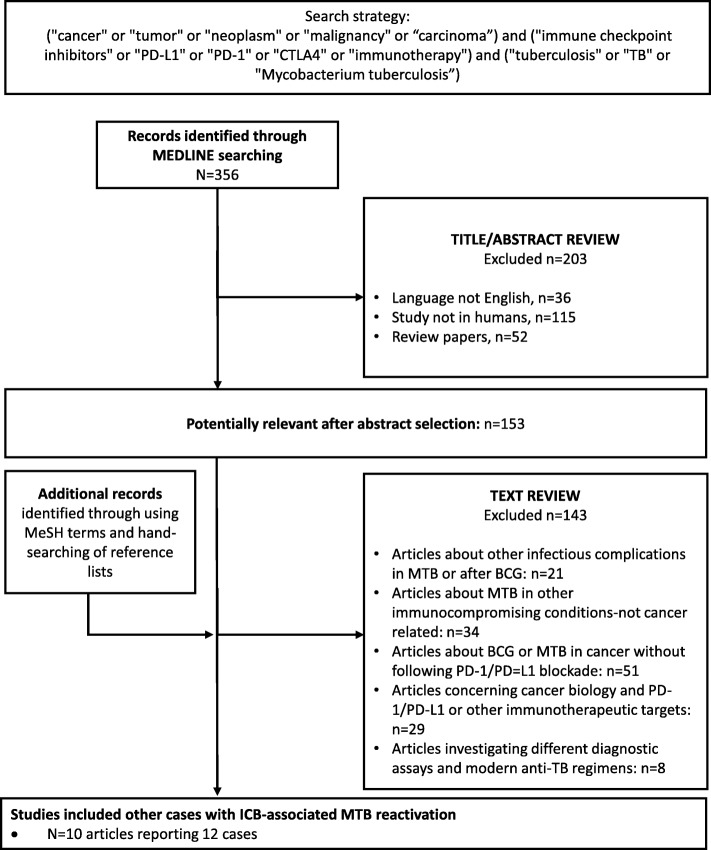
Flow diagram of literature search strategy

In order to identify other reported cases with ICB-associated MTB infection, we used the following terms for online search of PubMed: (1) terms suggestive of cancer (e.g., cancer, tumor, malignancy), (2) terms suggestive of immunotherapy (e.g., immune checkpoint inhibitors, PD-L1, PD-1, CTLA4, immunotherapy), (3) terms suggestive of tuberculosis (e.g., tuberculosis, TB, *Mycobacterium tuberculosis*). As restriction limits through the electronic search, we used English language and human-based studies. The full search strategy of the literature by the two independent reviewers, with the numbers of the records identified or excluded and the reasons of exclusions, is represented in Fig. 3, according to the PRISMA (Preferred Reporting Items for Systematic Reviews and Meta-analysis). A secondary expanded search was conducted by using Medical Subject Headings (MeSH) subjects and by hand-searching of reference lists from previous reviews to identify additional publications. For the cumulative presentation of findings among the identified case-reports, the 95%CIs of proportions were estimated by modified Wald method.

### Current evidence on MTB reactivation after ICPI treatment

We have identified ten reports that describe 12 cancer patients with active MTB infection after PD-1/PD-L1 blockade [37, 41–49]. Table 1 summarizes all these published cases with the addition of the two cases reported here. The reported patients were predominantly males (78.57, 95%CI:51.68–93.16%) and their age ranged from 49 to 87 years. Of 14 cases, 5 cases had advanced/metastatic non-small cell lung cancer (NSCLC) (35.71, 95%CI:16.18–61.40%), 5 had advanced/metastatic melanoma (35.71, 95%CI:16.18–61.40%), 2 had advanced/metastatic head and neck squamous carcinoma (HNSCC) (14.29, 95%CI:2.76–41.19%), 1 had metastatic Merkel carcinoma (7.14, 95%CI: < 0.01–33.54%), and 1 Hodgkin lymphoma (7.14, 95%CI:< 0.01–33.54%). For PD-1/PD-L1 inhibition nivolumab was used in 8 cases (57.14, 95%CI:32.55–78.66%), pembrolizumab in 5 cases (35.71, 95%CI:16.18–61.40%) and atezolizumab in the last case (7.14, 95%CI,< 0.01–33.54%). There are several points of interest. Only two of 14 patients (14.29, 95%CI,2.76–41.19%) that developed active MTB infection had required steroids or infliximab for any irAEs. Apart from the first case presented here only the case reported by Chu et al. [43] had received prednisolone 1 mg/kg for 1 month. Thus, cancer and ICB immunotherapy should be considered the probable basis for susceptibility to MTB in these cases. In most cases the initial diagnosis at onset of MTB-associated illness was cancer progression, due to shared findings of weight loss and new lung infiltrates. In the reported cases, it was not clear whether tuberculosis was primary or secondary to reactivation of latent disease. In all cases with available time-to-event information, the symptoms and signs of MTB infection developed within 6 months of PD-1/PD-L1 inhibition, suggesting latent tuberculosis reactivation. However, none of the patients had undergone any testing for LTBC before the initiation of immunotherapy despite MTB mortality rate of 28.57% (95%CI,11.34–55.03%). Regarding the treatment of ICB-associated MTB, all patients received rifampicin-containing regimens and cancer immunotherapy was temporarily discontinued in 5 cases (35.71, 95%CI,16.18–61.40%) while maintained in 3 cases (21.43, 95%CI,6.84–48.32%).

**Table 1:** Published cases of MTB reactivation in cancer patients treated with immunotherapy

First author, year	Age/sex (Origin)	Cancer type	ICB	Line (Duration)	Additional Immunosuppression (dose, duration)	Symptoms	MTB confirmation	Management	Outcome
Lee J, 2016 [41]	87/Male (Asian)	HL	Pembrolizumab	2nd (5 cycles)	None	Fever, fatigue and weight loss	Sputum culture (+)	Anti-MTB: 3-drug regimen ICB: Temporary Discontinuation	Complete remission of pulmonary MTB
Fujita K, 2016 [42]	72/Male (Asian)	Metastatic NSCLC	Nivolumab	2nd (8 cycles)	None	N/A	BAL culture (+), PCR(+)	Anti-MTB: N/A ICB: N/A	N/A
Chu YC, 2017 [43]	59/Male (Asian)	Metastatic NSCLC	Nivolumab	2nd (3 cycles)	Prednisolone (1 mg/kg, for 1 month)	Tamponade	Histology and Pericardial fluid culture (+)	Anti-MTB: N/A ICB: Maintenance	Complete regression of pericarditis
He W, 2018 [44]	65/Female (Asian)	Metastatic Melanoma	Pembrolizumab	1st (10 cycles)	None	Bloody sputum	AFB(+), PCR(+), Sputum Culture (+)	Anti-MTB: 4-drug regimen ICB:	Stop anti-MTB due to toxicity, second anti-MTB regimen Completion of 14 cycles of ICB
Jensen KH, 2018 [45]	56/Male (Caucasian)	Advanced NSCLC	Nivolumab	3rd (12 cycles)	None	Asymptomatic	AFB(+), PCR(+)	Anti-MTB medication, ICB: Discontinuation	N/A
Picchi H, 2018 [46]	50/Male (Caucasian)	Metastatic Melanoma	Pembrolizumab	1st (4 cycles)	None	Asymptomatic pleurisy	Histology and TST (+)	Anti-MTB: 4-drug regimen ICB: Maintenance	Complete regression of pleural effusion
	64/Male (Caucasian)	Metastatic NSCLC	Nivolumab	2nd (2 cycles)	None	Spinal cord compression	Histology, Bone culture (+), PCR (+)	Anti-MTB: ICB: Discontinuation	DOD: Rapidly after 2nd operation for spinal cord compression
Elkington PT, 2018 [47]	62/Female (Caucasian)	Metastatic Melanoma	Pembrolizumab	2nd (N/A)	None	Abnormalities in LFTs and an imaging lung lesion	Histology, BAL culture (+)	Anti-MTB: 4-drug regimen ICB: Temporary Discontinuation	Clinical improvement, normalization of LFTs and regression of the lung lesion
Tsai CC, 2019 [48]	49/Male (Asian)	Metastatic HNSCC	Nivolumab	2nd (6 cycles)	None	Fever and cough	AFB(+), PCR(+), Sputum Culture (+)	Anti-MTB medication, ICB: Discontinuation	DOD: 5 months after MTB diagnosis with bacterial pneumonia and ARF
Takata S, 2019 [49]	75/Male (Asian)	Metastatic NSCLC	Nivolumab	4th (15 cycles)	None	Fever, cough, and purulent sputum	AFB(+), Sputum Culture (+), PCR(+)	Anti-MTB: 4-drug regimen for 2 months, and 2-drug combination for 7 months ICB: Temporary Discontinuation	Nivolumab restarted after anti-MTB induction, reaching to PR after 46 cycles without relapse of MTB.
Barber DL, 2019 [37]	59/Male (Caucasian)	Metastatic HNSCC	Nivolumab	1st (3 cycles)	None	Asymptomatic	AFB(+), PCR(+), Sputum Culture (+)	Anti-MTB: 3-drug regimen ICB: Discontinuation	Patient worsened (supplemental oxygen, persistently febrile, and hypotensive) DOD: 2 months after initiation of ICB
	83/Male (Caucasian)	Metastatic MCC	Pembrolizumab	1st (12 cycles)	None	Asymptomatic	AFB (+)	Anti-MTB: 4-drug regimen ICB: Temporary Discontinuation	Anti-MTB: changed to 2-drug regimen due to elevated liver enzymes and completed in 9 months. MCC progressed and pembrolizumab restarted with resultant tumor reduction.
Current study, 2019									
Patient#1	76/Female (Caucasian)	Advanced Melanoma	Nivolumab+/−Ipilimumab	Adjuvan (8 cycles)	Methylprednisolone (32 mg daily for ~ 3 months) and infliximab (5 mg/kg for 3 doses)	Fever and cough	BAL culture (+), PCR(+)	Anti-MTB: 3-drug regimen ICB: Discontinuation	DOD: 3 days after anti-MTB initiation with ARF
Patient#2	85/Male (Caucasian)	Metastatic Melanoma	Atezolizumab+cobimetinib	1st (9 cycles)	None	Fever and cough	Sputum Culture (+)	Anti-MTB: 4-drug regimen ICB: Maintenance	Complete remission of pulmonary MTB, SD of melanoma and continuation of ICB

*Abbreviation*: *ICB* immune checkpoint inhibitor, *HL* Hodgkin Lymphoma, *HNSCC* head and neck squamous cell carcinoma, *NSCLC* non-small-cell lung cancer, *AFB* acid-fast bacilli, *BAL* bronchoalveolar lavage, *MTB* mycobacterial tuberculosis, *MCC* Merkel cell carcinoma, *SD* stable disease, *PR* partial response, *ARF* acute respiratory failure, *LFT* liver function tests, *DOD* date of death

## Clinical recommendations

Based on the synthesis of current evidence and our experience, we below address some emerging issues regarding the incidence and management of tuberculosis in oncological patients, and suggest clinical practice recommendations.

### Recommendations for LTBC screening in cancer patients

There is no clear recommendation whether cancer patients should be screened for LTBC and if positive, receive preventive chemoprophylaxis. According to 2018 WHO guidelines, cancer patients are not suggested for LTBC screening due to the lack of evidence [50], The USPSTF did not review evidence on screening of patients with any type of malignancy, since screening of these populations was already indicated prior to certain immunosuppressive medications, including chemotherapy or TNF–a inhibitors [51]. Current guidelines of the American Thoracic Society (ATS), the CDC and the Council of the Infectious Diseases Society of America (IDSA) recognize patients with leukemias and lymphomas, with head and neck or lung carcinoma as high-risk cases for MTB reactivation and subsequently recommend chemoprophylaxis when LTBC is documented in these groups [10, 52]. These guidelines are derived from studies between 1950s and 1970s and are limited by the absence of observation time at the estimation of relative risk. The risk for developing MTB is different among cancer types and continues to change over time as newer therapeutic strategies are developed. Targeted monoclonal antibodies and hematopoietic stem cell transplantation modified drastically the management of hematologic malignancies and produced diverse patterns of immunosuppression compared to therapies prior to the 1970s [53] while for HNSCC and lung cancer, new radiation modalities have decreased local tissue damage [54]. According to the National Institute for Health and Care Excellence (NICE), patients with LTBC having a hematological malignancy, having chemotherapy for any cancer type or having a gastrectomy for gastric cancer are at increased risk of developing tuberculosis, however, NICE does not provide specific screening and treatment recommendations for these groups [55]. Using Danish nationwide medical databases, Simonsen et al. concluded that the risk for active tuberculosis among cancer patients was significantly higher compared to age/sex-matched healthy controls after adjustment for other comorbidities [56]. The highest risks were observed in cancers of the aerodigestive tract, tobacco-related cancers, and hematologic malignancies [56]. Recently, Cheng et al. performed a systematic review and meta-analysis to quantify the risk of active MTB infection in cancer patients, including 23 studies with more than 300,000 patients [11]. Despite the methodological limitations, this study showed that individuals with hematologic, HNSCC, and lung cancers had a higher rate of developing active MTB compared to those without cancer and would benefit from targeted LTBC screening and chemoprophylaxis [11]. More specifically, in the six studies from United States published after 1980, the incidence rate ratio (IRR) was 26 for hematologic malignancies, 16 for HNSCC, 9 for NSCLC, and 4 for breast and other solid tumors [11]. For HNSCC and lung carcinoma, this increased risk may be confounded by other independent risk factors such as alcohol usage or smoking [57]. Dobbler et al. conducted another meta-analysis including 13 studies with more than 920,000 patients to estimate further the IRR of tuberculosis for patients with solid and hematologic malignancies compared to the general population [10]. In this study, lung (IRR = 6.14; 95%CI:1.97–19.20), gastric (IRR = 2.63, 95%CI:1.96–3.52), breast (IRR = 2.17; 95%CI:1.98–2.38) and colon cancer (IRR = 2.00, 95%CI:1.16–3.43), had a statistically significant greater IRR of developing MTB infection, instead of liver cancer that did not reach significance (IRR = 2.02; 95%CI:0.83–4.91) [10]. Gastric cancer had not a remarkably different IRR from other solid cancers, although it is often treated with gastrectomy and characterized by malnutrition [58], an independent risk factor for tuberculosis [59]. The IRR for MTB in patients with hematological malignancies (IRR = 3.53; 95%CI:1.63–7.64) was moderately higher compared to patients with solid tumors (IRR = 2.25; 95%CI:1.96–2.58).

Taken together, the aforementioned results support screening for LTBC among patients with hematologic malignancies, HNSCC, and lung cancer, based on the substantially increased incidence of active MTB in these groups. However, in patients with other solid tumors, screening for LTBC is not routinely performed and a risk-stratified approach should be proposed. First, the risk of MTB in these types of cancer is significantly lower compared to other immunocompromised groups, such as patients with HIV (RR:50–110) [47, 48], contact with individuals with active MTB (RR:10.4) [49], patients with chronic renal failure (RR:7.8) [5] and patients being treated with TNF-a inhibitors (RR:1.8–29.3) [50]. Although, the reactivation of MTB can occur at any time after initial infection [3], the estimated cumulative lifetime risk for developing active MTB is calculated by IRR, and this time-dependent parameter guides decisions about LTBC screening and chemoprophylaxis. For example, in chronic conditions with moderate effect upon life expectancy, such as diabetes and chronic renal failure, the potential risk is expected to last a whole lifetime. In contrast, the short-term immunosuppression induced by adjuvant treatment in earlier cancer stage, and the poor prognosis in metastatic stage, provide a reduced risk for developing active MTB infection. There is not a single threshold-risk of MTB (an IRR cut-off) over which to establish systematic screening and treatment for LTBC, in cancer patients, independent of other risk factors. The potential harms and benefits of LTBC treatment due to drug interactions or toxicities will also need to be weighed on an individual basis [60]. Among patients at low risk for hepatotoxicity (mainly due to isoniazide), LTBC testing is suggested for cases with an expected 5-year survival > 25% while among patients at increased risk for hepatotoxicity, LTBC testing is suggested for individuals with an expected 5-year survival > 50% [10, 11]. All these aspects including the type of cancer, the exposure to mycobacterium, the expected prognosis, the host comorbidities and the possible drug toxicities should be taken into account at the time of consideration for LTBC screening.

### Recommended tests for LTBC screening

Two screening tests for LTBC are currently used: a) the tuberculin skin test (TST), and b) the blood test of interferon-gamma release assay (IGRA). The TST requires intradermal placement of tuberculin purified protein derivative and interpretation of skin erythema and induration response 48–72 h later (for palpable swelling). Both T-SPOT.TB (Oxford Immunotec Global) and QuantiFERON-TB Gold In-Tube (Qiagen) are currently approved IGRAs and require just a blood sample for results within 8 to 30 h [51]. Despite this many patient factors and health system parameters may influence the selection of a screening test [61], guidelines by WHO, USPSTF, ATS, CDC and IDSA support testing with IGRA over TST for diagnosis of LTBC in individuals with low-to-intermediate risk of progression to active disease, and either IGRA or TST or dual testing (if first one is negative) at highest risk of developing active MTB [51, 62]. Before consideration of usage of TNF-a blocking agents, IGRA is preferable to TST because of lower false-positive results in patients treated with corticosteroids and/or with a previous BCG vaccination [63]. However, after reviewing 19 studies in immunosuppressed patients, Hasan et al. found that TST and IGRA were of equal value for LTBC screening [12].

### Exclusion of active tuberculosis on a cancer patient

After a positive IGRA or TST and prior to LTBC treatment, all patients must be evaluated to rule out active tuberculosis and to minimize the risk of drug resistance associated with anti-tuberculosis monotherapy. The evaluation includes clinical history, physical examination, and chest radiograph and respiratory sampling. The exclusion of active MTB infection remains a diagnostic challenge in an oncologic patient, since many symptoms (such as cough > 2 weeks’ duration, fevers, night sweats, weight loss and new interstitial infiltrates) can be due to either cancer progression or infection. Patients with relevant clinical manifestations and/or abnormal chest radiograph should submit 3 sputum specimens (obtained via cough or induction at least 8 h apart and including at least one early-morning specimen) for acid-fast bacilli (AFB) smear, mycobacterial culture, and nucleic acid amplification testing. All unexpected suspicious lung lesions depicted by imaging should be investigated or biopsied, if possible. As outlined by two older studies, active tuberculosis occurred, concurrently or soon after the tumor diagnosis, in more than half of patients with HNSCC or lung cancer [64, 65]. This active incidence of MTB remains elevated for the first year after cancer diagnosis and treatment initiation, and after that, declines over time [56, 66]; for example in hematological malignancies, from 12.01% (95%CI:10.81–13.30) in the first 6 months, reduces to 2.70% (95%CI:2.12–3.39) after 24 months [66]. Although the initially closer follow-up might result in an overestimation, it is clear that the presentation of cancer is a significant factor in the risk for developing active tuberculosis.

### Targeted LTBC screening before immunotherapy

In developing countries with high prevalence of MTB, the limited use of ICBs and the short follow-up may lead to underreporting of the real risk in the current literature, while in developed countries, personalized therapeutic approaches based on the cancer stage, tumor molecular profile and expected prognosis makes it more difficult to differentiate between the risk arising from cancer per se and that arising from a specific treatment option. Although therapeutic advances and prolongation of survival in melanoma patients may influence the lifetime risk of developing or reactivating MTB infection, it is not clear whether the addition of immunotherapy or other anticancer treatments significantly increase the risk associated with cancer itself. A direct comparison of MTB rates between ICB-treated and non-ICB treated cancer patients is not feasible, since available data are limited. To enlighten as much as we can any difference, we present here the population-based tuberculosis rates rather than standardized individual risk for person-years, which is the standard approach. An older report by Memorial Sloan-Kettering Cancer Center described that the incidence of MTB was highest in patients with hematologic malignancies with a notification rate greater than 200 cases per 100,000 persons, > 2/1000), a rate similar to that of patients with HIV infection or with recent mycobacterial exposure. The incidence varied significantly according to the country of birth [67]. Among patients with solid tumors, the overall rate of MTB reactivation was 39 cases per 100,000 persons (0.39/1000) and varied significantly between US-born and non-US-born patients. Patients with HNSCC had a substantially increased MTB rate (135 cases per 100,000 persons, 1.35/1000) that was unrelated to country of birth. In this large study, the mortality rate of MTB-infected cancer patients was similarly high (25%) with this one described here in ICB-treated patients (28.57%) and all cases died within 3 months of MTB diagnosis [67]. According to the French prospective registry managed by Gustave Roussy cancer center the rate of tuberculosis among cancer patients receiving anti-PD1/PD-L1 agents was estimated about 1/1000 patients [46]. In our institution we have treated with ICBs, approximately 500 melanoma patients in the adjuvant or metastatic setting, either in a clinical trial or after the regulatory approval of immunotherapy. Among this ICB-treated melanoma population, we diagnosed the 2 above-mentioned cases with MTB reactivation (~ 2/500). With the assumption that our patients had been exposed for the same duration in immunotherapy with those treated at Gustave Roussy, the estimated Poisson rates for the two populations did not differ significantly. Notably, this high observed MTB rate could not be justified only by the WHO and ECDC country-specific data for USA (3.1 per 100,000), France (9 per 100,000), and Greece (4.5 per 100,000), even if one takes into account the underreporting of diagnosed cases, in Greece and the significant immigration from endemic regions (Iraq, Afghanistan, India, Africa etc.).

Given that an anti-PD1 agent might favor tuberculosis reactivation, although without strong and direct evidence, Picchi et al. [46], suggested screening for LTBC with an IGRA in all cancer patients before initiation of immunotherapy. However, the cost of such strategy may not be affordable and the clinical benefit of treating all positive cases remains uncertain [68]. At this point, we propose that a targeted LTBC screening before the administration of ICPIs should be considered, especially in cancer individuals with one or more independent risk factors (diabetes, chronic renal failure, possible exposure to MTB or further immunosuppression). Apart from individual risk factors, all candidates for adjuvant immunotherapy (e.g. cases with melanoma stage III), which are considered free of cancer, should be tested for LTBC to ensure that they will not experience at the close future any complication by reactivated MTB. Since there is no knowledge whether LTBC testing is affected by the ICPIs, it is generally suggested to perform this screening before the administration of immunotherapy. Recently, ESCMID Study Group Consensus supported LTBC screening before starting of any anti-TNF-α therapy as the standard of care, followed by appropriate anti-tuberculosis therapy [69].

Cancer patients treated with immunotherapy develop a continuously expanding spectrum of irAEs, and most of them require steroids and/or anti-TNFa agents for management when these become severe. Based on a recent review of infectious complications in melanoma patients treated with ICBs, the major risk factor for serious infections was the use of additional immunosuppressive agents, reaching an incidence of 13.5% in patients for corticosteroids or infliximab but only 2% in those who did not [9]. In agreement with our observations, infections occurred during the first 6 months after initiation of ICBs in 79.6% of patients [9]. Cancer patients on dual immunotherapy with nivolumab and ipilimumab or on combination with immuno- and chemo-therapy present higher incidence of irAEs (requiring steroids or steroid-sparing agents) and deeper degree of immunomodulation (developing more often ICB-associated infections) [6]. These patients belong in the high-risk group for MTB reactivation and need to be screened for LTBC before initiation of immunotherapy. However, in some cases the critical status of patients due to their disease or their severe irAEs may not permit waiting for LTBC testing results and the anticancer treatment is rashly prioritized.

The treatment of LTBC includes 4 months of rifampin or 9 months of isoniazid, or 3 months of once weekly directly observed therapy with isoniazid plus rifapentin [70]. However, no available data exist regarding LTBC chemoprophylaxis in PD-1/PD-L1 blockade and the therapeutic strategy here is based on evidence from TNF-a inhibition. According to findings from patients receiving anti-TNF agents, a 4-week chemoprevention with isoniazid reduces significantly the risk of developing active MTB [17]. Although more recent studies and CDC guidelines have suggested that LTBC treatment can start any time (even concurrent) with anti-TNF-a administration [71, 72], we propose that treatment prior to beginning anti-PD-1/anti-PD-L1 antibodies (e.g. 2 weeks) is more reasonable in order to assure patient tolerance of anti-tuberculosis prophylaxis. Patients should be monitored monthly for clinical signs of hepatitis during therapy for LTBC.

### Management of MTB reactivation during immunotherapy

To date, there is no evidence-based strategy for treating re-activated MTB during immunotherapy. Despite the theoretical benefit of PD-1/PD-L1 blockade in treating various infections, as well as tuberculosis [73, 74], it is generally supported that ICBs should be withheld during active infection, because of the possibility of an exaggerated inflammatory response. However, the exact timing for safe resumption of immunotherapy after initiation of anti-tuberculosis treatment remains to be defined. Adopting the same strategy with LTBC prophylaxis, a 2-weeks interval with anti-tuberculosis treatment is also suggested before re-starting of immunotherapy. In cases with concurrent initiation of anti-tuberculosis treatment and maintenance of anti-PD-1/PD-L1 therapy, close monitoring is required to detect overlapping toxicities, especially liver dysfunction.

## Conclusions

It is accepted that ICBs may have infectious complications, indirectly as a consequence of the need for corticosteroids or TNF-a inhibitors to control irAEs associated with ICB therapy. Tuberculosis may be an exception to this rule, as the majority of reported cases from the literature and our experience were receiving neither corticosteroids nor TNF-a inhibitors when their reactivation was documented. Therefore, MTB reactivation may represent a direct complication of immunotherapy, although more data are needed to unequivocally establish this. The exact mechanism of increased susceptibility to MTB following administration of ICBs is not yet known. Preclinical data recognize a crucial role of PD-1/PD-L1 blocking in T cell exhaustion, evasion of immune surveillance and development of active tuberculosis. However, in clinical practice, the management of M. tuberculosis among cancer patients receiving ICBs presents challenges. Cancer itself is an independent risk factor for developing active MTB infection. This generally occurs early in the course of disease, and cancer progression is the most common misdiagnosis when constitutional symptoms such as weight loss and fever, common with active MTB, are developed. Thus, before changing treatment for a supposed disease progression or initiating corticosteroids for a suspected irAE, all cancer patients with appropriate symptomatology should be tested for tuberculosis and checked for any previous exposure to MTB, and other risk factors. The prompt diagnosis of a mycobacterial infection, even in a subclinical stage, is essential to avoid later potentially morbid exacerbation. Given that inhibition of PD-1/PD-L1 pathway may favor tuberculosis reactivation, targeted screening for LTBC is suggested before initiation of an ICPI, especially in cancer subjects with additional independent risk factors (e.g. host comorbidities, exposure to MTB endemic regions, and immunosuppression). The preferred diagnostic modality (e.g. a single test or combination of TST and IGRA) for LTBC screening in these patients has not been clearly defined. In addition, no available data exist for the management of latent or active tuberculosis during PD-1/PD-L1 blockade; for this reason, therapeutic guidelines are adopted from the management of patients receiving TNF-a inhibition. In general, in case of active tuberculosis, ICPIs are temporarily withheld, any further immunosuppression is discontinued and anti-tuberculosis treatment is timely initiated. Also, in patients diagnosed with either active or latent tuberculosis, it is not clear how long after the corresponding anti-TB treatment ICPIs should be safely resumed or initiated, with a duration of 2–4 weeks to be suggested. The continuously expanded implementation of IPCIs in cancer treatment requires resolving of these challenges by upcoming research data in order to maximize the clinical benefits of immunotherapy uninterruptedly and safely.

## Data Availability

Data supporting the recommendations of this article are included within the reference list. Please contact corresponding author for any further data request or supplementary information.

## Abbreviations

AFB: Acid-fast bacilli
ATS: American Thoracic Society
BAL: Bronchoalveolar lavage
CDC: Center for Disease Control and Prevention
CRP: C reactive protein
CT: Computed tomography
HNSCC: Head and neck squamous cell carcinoma
ICBs: Immune checkpoint inhibitors
IDSA: Infectious Diseases Society of America
IFN: Interferon
irAEs: Immune-related adverse events
IRIS: Immune reconstitution inflammatory syndrome
IRR: Incidence rate ratio
LTBC: Latent tuberculosis infection
MTB: Mycobacterial tuberculosis
NICE: National Institute for Health and Care Excellence
NSCLC: Non-small cell lung cancer
PCR: Polymerase chain reaction
Th1: T-helper type 1
TNF-a: Tumor necrosis factor-alpha
TST: Tuberculin skin test
USPSTF: US Preventive Services Task Force
WHO: World Health Organization
